# Diagnostic yield and safety of biopsy guided by electromagnetic navigation bronchoscopy for high‐risk pulmonary nodules

**DOI:** 10.1111/1759-7714.13930

**Published:** 2021-03-21

**Authors:** Ju Hyun Oh, Chang‐Min Choi, Seulgi Kim, Woo Sung Kim, Hee Sang Hwang, Se Jin Jang, Sang Young Oh, Mi Young Kim, Jae Cheol Lee, Wonjun Ji

**Affiliations:** ^1^ Department of Pulmonary and Critical Care Medicine, Asan Medical Center University of Ulsan College of Medicine Seoul South Korea; ^2^ Department of Oncology, Asan Medical Center University of Ulsan College of Medicine Seoul South Korea; ^3^ Department of Pathology, Asan Medical Center University of Ulsan College of Medicine Seoul South Korea; ^4^ Department of Radiology, Asan Medical Center University of Ulsan College of Medicine Seoul South Korea

**Keywords:** Biopsy, electromagnetic navigation bronchoscopy, learning curve, peripheral lung nodule, safety

## Abstract

**Background:**

Electromagnetic navigation bronchoscopy (ENB) is a useful method to obtain tissue for peripheral lung nodules. We aimed to understand the diagnostic yield and safety profile in high‐risk pulmonary nodules that cannot be accessed by percutaneous transthoracic needle biopsy.

**Methods:**

In this single‐center retrospective study, we reviewed patients who underwent ENB for high‐risk pulmonary nodules. All procedures were performed under moderate sedation using intravenous midazolam and fentanyl.

**Results:**

A total of 100 pulmonary nodules in 90 patients were subjected to ENB between October 2018 and May 2020. The median age of the study population was 66 (59–73). The mean diameter of the lung nodules was 27.9 mm. The diagnostic yield of ENB‐guided biopsy was 53.0%. Although the nodule size (odds ratio: 1.055, *p* = 0.007) and positive bronchus sign (odds ratio: 2.918, *p* = 0.020) were associated with the diagnostic yield during univariate analysis, nodule size was the only independent variable on the multivariable analysis. Interestingly, the diagnostic yield showed an upward trend after 60 cases, from 45%–65%. Procedure‐related complications were reported in 16 cases; among these, pneumothorax occurred in three cases, and four cases experienced moderate bleeding. No instance of major bleeding or death was linked to ENB‐guided biopsy.

**Conclusion:**

ENB‐guided biopsy for high‐risk pulmonary nodules demonstrated an acceptable diagnostic yield and good safety profile. Moreover, the diagnostic yield was associated with nodule size and procedure experience.

## Graphical abstract

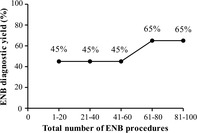

In this single‐center retrospective study, we reviewed a total of 100 pulmonary nodules in 90 patients who underwent ENB for high‐risk pulmonary nodules under moderate sedation. The diagnostic yield of ENB‐guided biopsy was 53.0% and showed an upward trend after 60 cases, from 45%–65%. The diagnostic yield was associated with nodule size and the operator's experience with the procedure. Procedure‐related complications were reported in 16 cases without major bleeding events or death. ENB can be used for high‐risk patients because of an acceptable diagnostic yield and a good safety profile.

## INTRODUCTION

The early detection of lung nodules is important in diagnosing curable lung cancer.^1^, ^2^ With the growing use of chest computed tomography (CT), peripheral lung nodules are being detected in increasing numbers. In the National Lung Screening Trial, 39.1% of participants in the low‐dose chest computed tomography (LDCT) screening group had at least one positive screening result.^3^

Several techniques are currently used to approach peripheral lung nodules, yet selecting the proper modality for evaluation of suspicious lung nodules can be a challenge for physicians.^4^ According to the American College of Chest Physicians lung cancer guidelines, the least invasive approach is recommended after considering factors such as the lesion size, nodule location, procedure‐related complications, patient comorbidities, and operator expertise.^5^ A flexible bronchoscopic biopsy is useful for visible endobronchial lesions but has shown rates of diagnostic yield between 14% and 69% depending on the lesion size and location.^5^, ^6^, ^7^ In the context of peripheral lung nodules, percutaneous transthoracic needle biopsy (PCNB) has been widely adopted and has sensitivity reaching 90%. However, the relatively high risk of complications limits the widespread use of this approach.^8^, ^9^

Electromagnetic navigation bronchoscopy (ENB) has recently been introduced to increase diagnostic yield while limiting complication risk.^10^, ^11^, ^12^, ^13^, ^14^ However, the diagnostic yield and safety profile of ENB for high‐risk pulmonary nodules that cannot be assessed by percutaneous methods are not well‐researched. Most of the previous research reported the results of patients who underwent ENB procedures with general anesthesia. In this study, we assessed the diagnostic yield, accuracy, and safety of ENB for high‐risk pulmonary nodules conducted with moderate sedation and analyzed the factors that may influence the ENB results. We also evaluated changes in the diagnostic yield that occurred with growing operator experience over time.

## METHODS

### Study population

This was a retrospective, single‐center study involving subjects who underwent ENB at Asan Medical Center, Seoul, Republic of Korea, from October 2018 to May 2020. Eligible participants included candidates for the biopsy of lung lesions detected previously on chest CT scan. All candidates were assessed for possible PCNB with respiratory physicians and radiologists. A percutaneous procedure was not recommended because of the inaccessible location of lung nodules or the high risk of procedure‐related complications. Patients in whom PCNB failed to obtain proper tissues were also included. We defined the nodules at high risk with the percutaneous approach as “high‐risk pulmonary nodules” in this study. The study protocol was approved by the institutional review board of Asan Medical Center (approval no. 2020‐1009).

### Procedures and clinical measurements

Clinical data, including age, sex, smoking history, pulmonary function test results, and radiologic findings, were retrieved from medical records. Nodule size and the presence of bronchus sign on chest CT scans were evaluated before ENB. If a bronchus sign was present, it was classified as concentric or eccentric according to its location in the target nodule. The operator took ENB‐related measurements during the procedure. The maximum standard uptake value (SUV_max_) of lesions per fluorodeoxyglucose positron emission tomography/computed tomography (FDG PET/CT) before ENB was also recorded. We sorted ENB results into positive, intermediate, or indeterminate groups according to the description in a previous study.^12^ Procedure‐related complications were classified according to type and severity. Pneumothorax was confirmed by a post‐procedure chest X‐ray, and the need for chest tube drainage was additionally described.

If a bleeding event stopped spontaneously, it was defined as minor, whereas moderate bleeding required cold saline and topical epinephrine. Severe bleeding was designated when a blood transfusion was required.^15^ The final diagnosis was confirmed by surgery or additional CT follow‐up after at least 3 months. The primary endpoint of this study was the diagnostic yield of ENB‐guided biopsy, whereas the secondary endpoints were the safety profile, factors associated with the diagnostic yield, and change in the diagnostic yield according to the accumulation of procedure experience.

### ENB process

**FIGURE 1:**
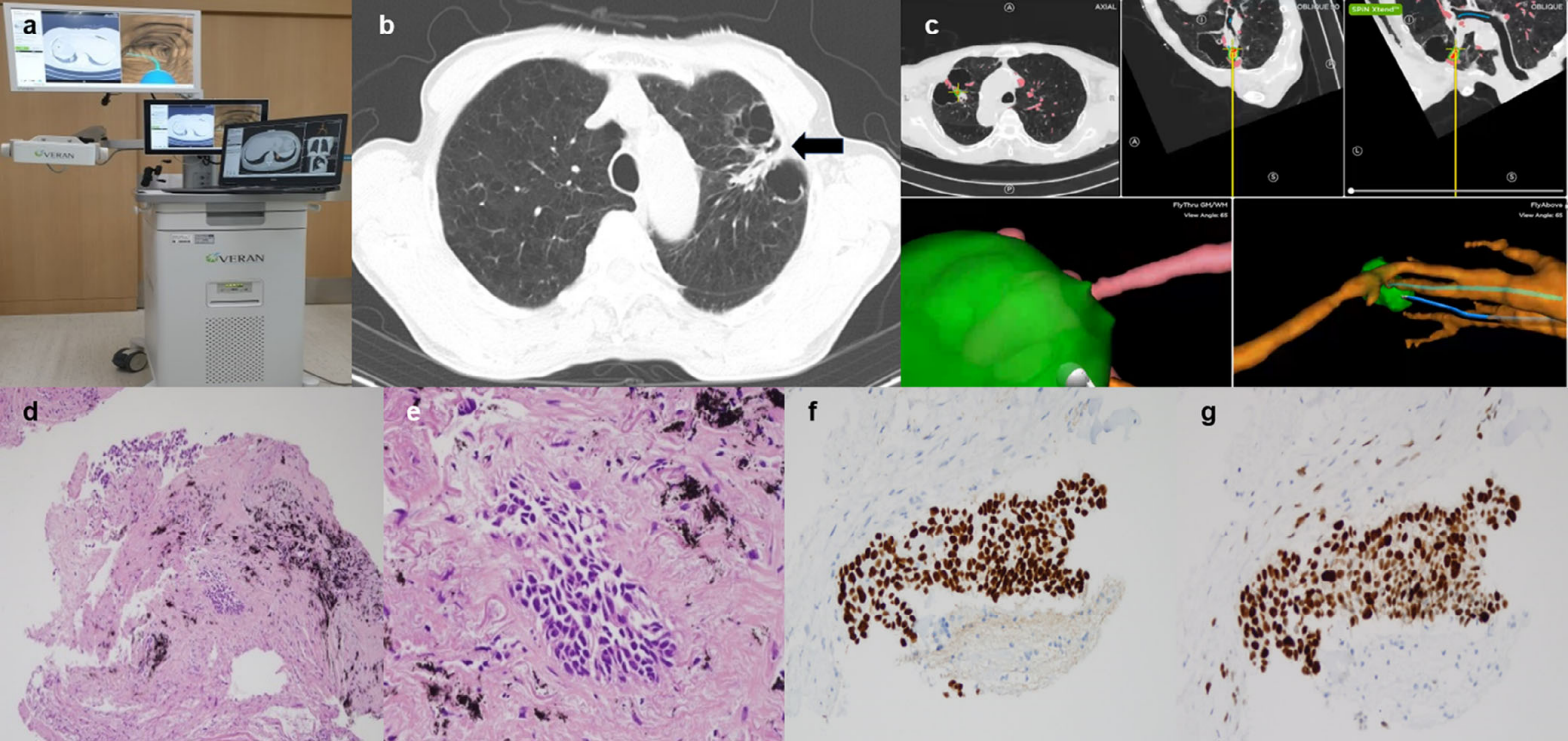
(a) SPiN thoracic navigation system. (b) Chest CT image identifying ill‐defined consolidation at the cystic lesion periphery on the left upper lobe (arrow). (c) Screen image during ENB‐guided biopsy with forceps. (d–g) ENB‐guided biopsy specimen demonstrating squamous cell carcinoma. Atypical cell clusters in fibro‐elastic stroma were found in specimen (×100 (d) and ×400 (e)). Immunohistochemial staining showed positive for p40 (f) and overexpression of p53 (g)

On the day of the procedures, all patients underwent chest CT per the protocols recommended by available commercial navigation platforms to reconstruct a virtual airway route.^16^ In the planning phase, the target lesion was identified and the closest immediate airway to the lesions was determined. All patients received 2–3 mg of midazolam and 50 μg of fentanyl intravenously at the onset of the ENB procedure. All procedures were performed by a single interventional pulmonologist (W.J.) at Asan Medical Center. We used the SPiN Thoracic Navigation System (SYS‐4230 K; Veran Medical, St. Louis, MO) for ENB and a bronchoscope with an outer diameter of 4 mm (P260F) or 6 mm (1 T260) (both Olympus Corporation, Tokyo, Japan) (Figure 1). We primarily used 6 mm scopes to explore inside the bronchus and determine the possibility of approaching the lesion. ENB guided biopsy was done with a 6 mm scope if it was sufficient to access the target lesion and if not, we used a 4 mm scope to get as close as possible to the lesion. We did not use additional equipment, such as radial endobronchial ultrasound (R‐EBUS), fluoroscopy, and rapid on‐site examination (ROSE). Most of the biopsies were performed via forceps, and needle aspiration was applied on the lesions that had unclear bronchus signs but were adjacent to the bronchus.

### Statistical analysis

Categorical variables are shown as numbers and percentages, and continuous variables are presented as means ± standard deviation or median (interquartile range). Univariate and multivariate logistic backward stepwise regression models were established to analyze factors affecting the diagnostic yield. Variables with a *p*‐value of <0.1 on univariate analysis were entered into the multivariate analysis. Diagnostic yield was defined as the percentage of definite malignant or benign diagnosis confirmed by ENB‐guided biopsy among the total targeted nodules.^12^ A *p*‐value of <0.05 was considered significant. Statistical analyses were performed using the software program Statistical Package for the Social Sciences version 21.0 (IBM Corporation, Armonk, NY).

## RESULTS

### Baseline characteristics of participants

A total of 98 patients underwent ENB procedures. We failed to approach the lesions by ENB in seven of these patients, and tissue from endobronchial lesions was obtained by bronchoscopy before ENB in one patient. Ninety patients were analyzed, and the ENB success rate was 93.5% (100/107 nodules). The clinical characteristics of the study participants are described in Table 1. The median age of the included patients was 66 years (interquartile range = 59–73 years), and 55 (61.1%) patients were male. A pulmonary function test was conducted in 81 subjects, with mean values of forced vital capacity and forced expiratory volume in 1 second of 86.1% and 80.7%, respectively. In the majority of cases (63%), ENB was performed because of the high bleeding risk associated with PCNB because of nearby vascular structures, whereas 16 cases presented a risk of pneumothorax because of emphysema.

**TABLE 1:** Baseline characteristics of the study participants

	Number (%)
Total participants	90
Age in years, median [range]	66 [59–73]
Male sex	55 (61.1)
Smoking	
Never	42 (46.7)
Ex‐smoker	34 (37.8)
Current smoker	14 (15.6)
Pulmonary function (n = 81)	
FVC, % pred, mean ± SD	86.1 ± 14.0
FEV1, % pred, mean ± SD	80.7 ± 20.6
DLco, % pred, mean ± SD	74.6 ± 24.9
Cause of ENB (n cases = 100)	
Emphysema	16 (16.0)
Vascular structure	63 (63.0)
Inaccessible	7 (7.0)
No diagnosis by PCNB	7 (7.0)
At high‐risk of pneumothorax and bleeding	7 (7.0)
Intravenous sedation	90 (100)
ENB procedure time, mean ± SD, min	12.9 ± 6.0
Total procedure time, mean ± SD, min	17.4 ± 7.0

*Note*: Data are presented as number (%), mean ± SD, or median (interquartile range).

*Abbreviations*: DLco, diffusing capacity of the lung for carbon monoxide; FEV_1_, forced expiratory volume in 1 second; FVC, forced vital capacity; IV, intravenous; PCNB, percutaneous needle biopsy; SD, standard deviation.

### Baseline characteristics of pulmonary nodules

An ENB‐guided biopsy was performed on a total of 100 nodules (Table 2); 28.0% were located in the right upper lobe. The median size of the nodules was 27.9 mm, and most lesions were solid type (55%). Further, 71 nodules presented a bronchus sign on the CT scan, including 55 nodules with a concentric type of bronchus sign. The SUV_max_ of the target lung lesion on FDG PET/CT was 6.1. Forceps were used for biopsy in 63 nodules, and the combination of forceps and a needle was used for 34 nodules.

**TABLE 2:** Baseline characteristics of pulmonary nodules

	Number (%)
Total nodules	100
Location	
Right upper lobe	28 (28.0)
Right middle lobe	9 (9.0)
Right lower lobe	19 (19.0)
Left upper lobe	21 (21.0)
Left lower lobe	23 (23.0)
Size, mm ± SD	27.9 ± 13.7
Type	
Solid	55 (55.0)
Ground‐glass opacity	5 (5.0)
Partially solid	33 (33.0)
Consolidation	7 (7.0)
Bronchus sign present	71 (71.0)
Concentric	55 (55.0)
Eccentric	16 (16.0)
Metabolic activity in PET, mean SUV_max_ ± SD (n = 76)	6.1 ± 5.1
Distance from visceral pleura, mm ± SD	13.7 ± 12.8
Biopsy tool used	
Forceps	63 (63.0)
Needle	3 (3.0)
Forceps + needle	34 (34.0)
Number of biopsy attempts per lesion	6.68 ± 2.10

*Abbreviations*: SD, standard deviation.

### Factors associated with the diagnostic yield

**FIGURE 2:**
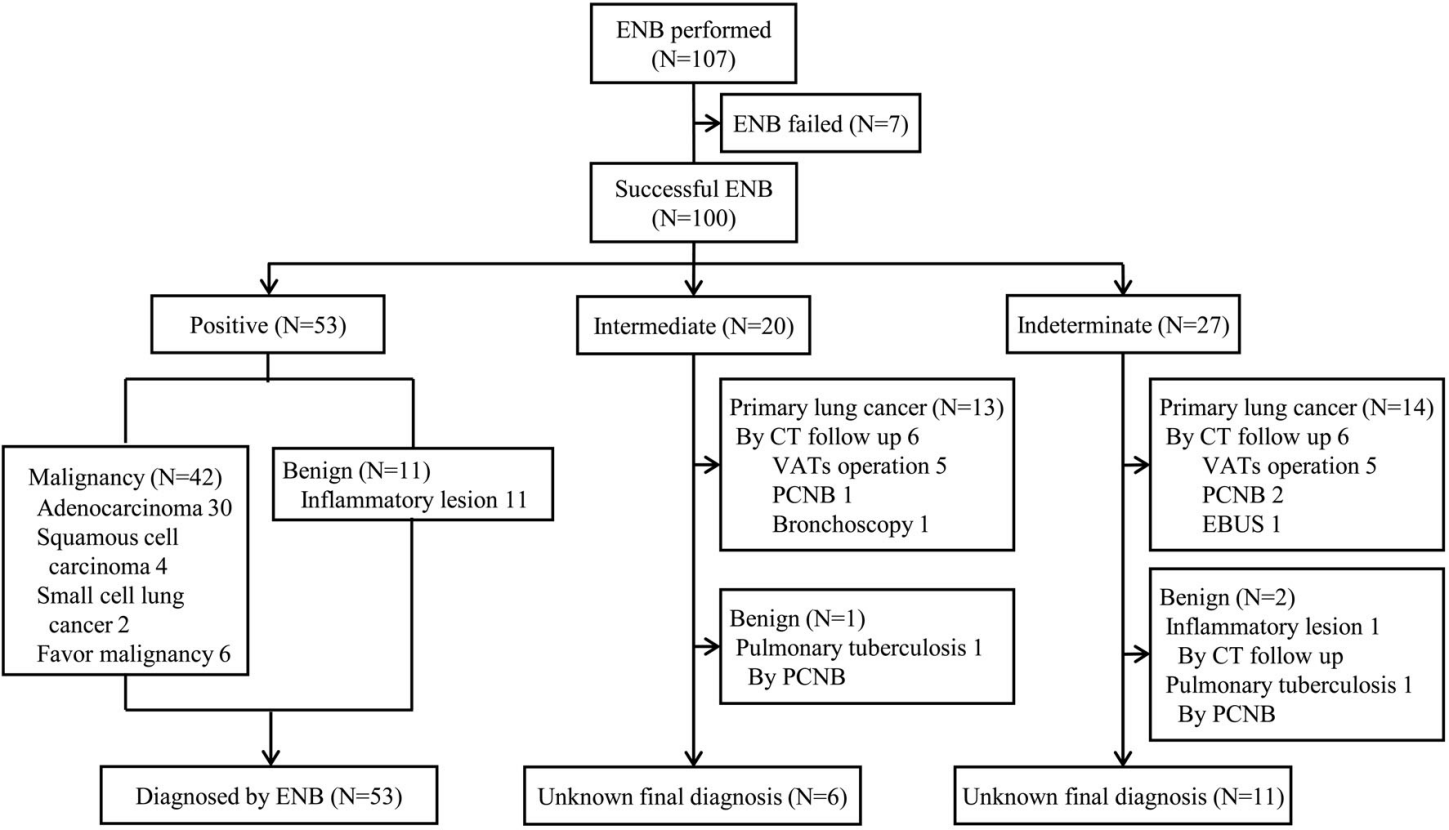
Flow chart of final diagnosis of ENB performed nodules

Figure 2 presents the final diagnosis of all nodules. The total diagnostic yield was 53% (53/100), and the overall accuracy was 63.9% (53/83). The sensitivity for malignancy was 60.9% (42/69). In the univariate analysis, nodule size and positive bronchus sign were associated with diagnostic yield. However, regardless of the bronchus sign type, only nodule size was an independent factor affecting the diagnostic yield in the multivariable analysis (Table 3). We identify the diagnostic yield according to each factor analyzed in the univariate and multivariate analysis in Tables S1 and S2. Three cases among the 100 nodules had a final diagnosis of lymphoma. Among them, one case (1/3 cases; 33.3%) was chronic lymphoplasmacytic inflammation with sclerosis in the ENB biopsy, suggesting lymphoma confirmed by surgical resection.

**TABLE 3:** Univariate and multivariable analysis of factors associated with the diagnostic yield

	Univariate analysis		Multivariate analysis	
	OR (95% CI)	*p*‐value	OR (95% CI)	*p*‐value
Upper lobe lesion	1.180 (0.538–2.591)	0.680		
Nodule size, mm	1.055 (1.015–1.097)	0.007	1.051 (1.009–1.094)	0.017
Solid type nodule	0.741 (0.118–4.636)	0.748		
Bronchus sign	2.918 (1.184–7.188)	0.020		
None	1 (ref)			
Concentric	2.850 (1.117–7.272)	0.028	1.720 (0.610–4.845)	0.305
Eccentric	3.167 (0.890–11.266)	0.075	1.858 (0.471–7.329)	0.376
Metabolic activity, SUV_max_	1.006 (0.920–1.100)	0.900		
Distance from visceral pleura, mm	0.978 (0.947–1.009)	0.159		
Total procedure time, min	0.940 (0.882–1.001)	0.054	0.943 (0.0.882–1.008)	0.083
Biopsy device		0.754		
Forceps only	1 (ref)			
Needle only	0.455 (0.039–5.272)	0.490		
Forceps + needle	1.152 (0.498–2.663)	0.454		
Movement distance ≥ nodule size	2.432 (0.422–14.027)	0.320		

*Note*: Variables with *p* < 0.1 on univariate analysis were included in multivariate analysis.

*Abbreviations*: CI, confidence interval; OR, odds ratio.

### Diagnostic performance over time

**FIGURE 3:**
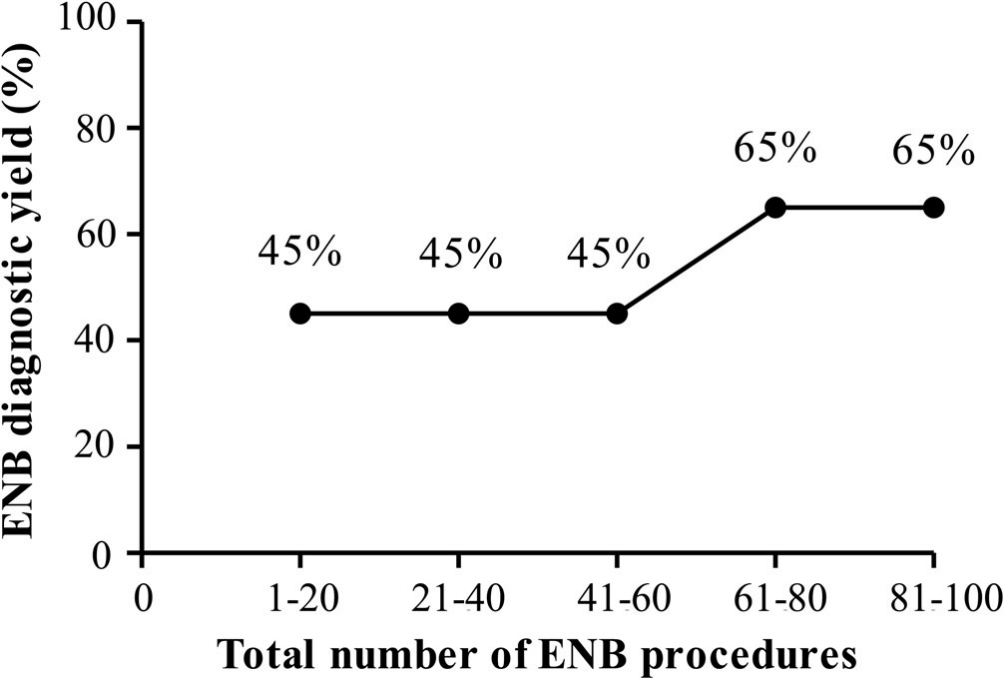
The learning curve of electromagnetic navigation bronchoscopy (ENB)

**FIGURE 4:**
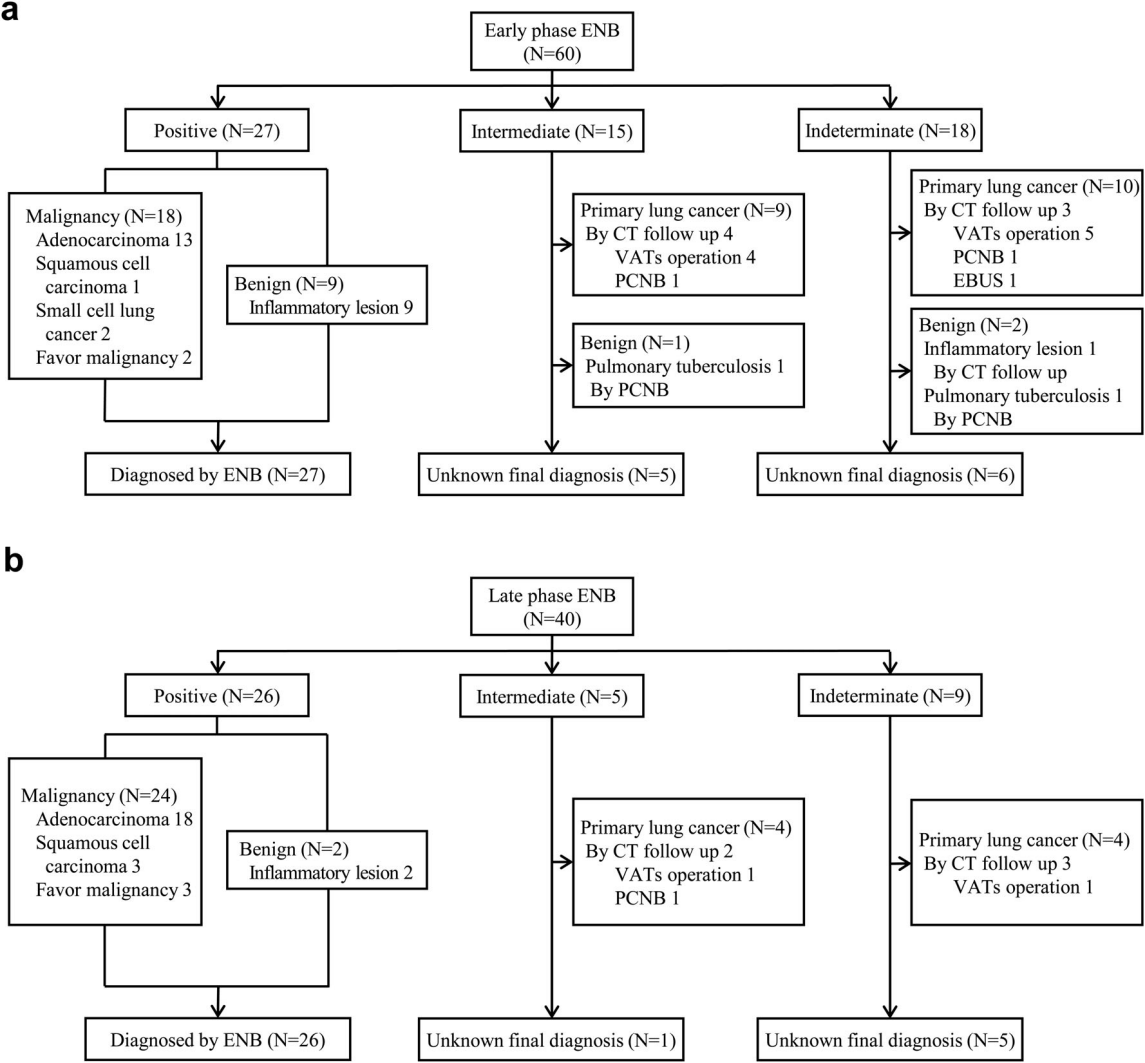
Flow chart of final diagnosis of ENB performed nodules in (a) early phase (1–60 cases) and (b) late phase (61–100 cases)

We divided the total number of ENB procedures into groups of 20 cases chronologically and examined the diagnostic yield of each section. Notably, the diagnostic yield was 45% for the first 60 procedures, but it gradually increased up to 65% for subsequent ones (Figure 3). We divided the total procedures into two phases: the early (1–60 cases) and late (61–100 cases) phases. Figure 4 shows the final nodule diagnosis in each phase. There were no significant differences in subject or nodule baseline characteristics identified between the phases, except for the mean SUV_max_ in PET and biopsy tools. The ENB procedure time was shorter in the late phase (61–100 cases) than the early phase (1–60 cases) (Tables S3 and S4 in the Supporting Information).

### Safety profiles

Procedure‐related complications occurred in 16 of 100 nodules. Bleeding (13%) was the most frequent complication, with a minor number of cases experiencing moderate bleeding (4%). Among the patients with moderate bleeding, one experienced respiratory failure, requiring intubation due to obstruction of the bronchus by a blood clot. Pneumothorax occurred in three patients, and one patient required chest tube insertion. No participant died of a procedure‐related complication (Table 4).

**TABLE 4:** Safety profile of ENB

Adverse event	Number (%)
Overall	16 (16)
Pneumothorax	3 (3)
Need for chest tube insertion	1 (1)
Total bleeding	13 (13)
Major	0 (0)
Moderate	4 (4)
Minor	9 (9)
Respiratory failure	1 (1)
Death	0 (0)

## DISCUSSION

In this retrospective study, the overall diagnostic yield of ENB was 53%, however, a positive trend of up to 65% was observed as the procedure experience increased. Nodule size was an important factor that significantly affected the diagnostic yield, whereas nodule type, location, and biopsy tool were not. Complications occurred in 16% of patients, but there were no procedure‐related deaths or untreatable, major complications.

Several studies investigating ENB's efficacy in diagnosing peripheral pulmonary nodules have reported variable diagnostic yields ranging from 69%–82.5%.^13^, ^17^ Although the diagnosis rate in this study was lower than that in previous studies, there are several considerations when interpreting these results. First, the type of sedation could affect ENB performance. Respiratory variation, coughing, and respiratory distress during the procedure might cause navigation errors, adversely influencing the diagnostic yield.^18^ In the present study, all patients breathed spontaneously under conscious sedation during the procedure. Because most previous ENB studies reported the results of patients undergoing the procedure with general anesthesia, they reported higher rates of diagnostic yield.^13^, ^14^, ^19^, ^20^ In a meta‐analysis of 15 ENB studies, general anesthesia during the procedure ensured significantly better diagnostic yields than conscious sedation (69.2% vs. 57.5%; *p* = 0.02).^12^ This suggests that how well the patient is sedated during ENB can greatly impact the diagnostic yield.

Second, we analyzed only patients with high‐risk lung nodules that could not be approached by PCNB. Previous studies included all pulmonary nodules, therefore, data on high‐risk nodules that are difficult to access by PCNB remain insufficient. Lung cancer occurs mainly around lesions that contain emphysema or honeycombing, especially in patients with chronic obstructive or interstitial lung disease.^21^, ^22^ In this case, it is impossible to perform PCNB on hard‐to‐access lesions even by ENB because of the risk of complications. Therefore, considering the characteristics of these high‐risk nodules, the relatively low diagnostic yield reported here as compared with prior studies could be explained. Finally, the study was the first retrospective report to use the SPiN Thoracic Navigation System in Asia, and the learning curve should be considered. Early ENB studies reported a wide range of diagnostic yields, from 49.0%–87.5%, suggesting that each center might have different diagnostic rates according to their procedure experience.^12^ In this study, we demonstrated that the diagnostic yield changes as the operator accumulates experience.

The current study identified nodule size as an important factor associated with diagnostic yield. In previous investigations, the bronchus sign was also reported as a significant variable affecting the diagnostic yield.^13^, ^23^ This trend was observed in the univariate analysis but was irrelevant in the multivariate analysis in this study. This might be because of the use of a needle and forceps in some cases. If the nodule was directly adjacent to the airway, even without the bronchus sign being present, a biopsy was performed successfully by transbronchial needle insertion. We suggest that using a needle rather than forceps for biopsy could help ensure an accurate diagnosis in the case of close airway nodules without a bronchus sign. Although previous research reported a lower diagnostic yield in lower‐lobe lesions affected by breathing movements,^24^ no significant difference was observed in our study. This might be because of the equipment, which provided real‐time respiratory movement tracking during the procedure based on inspiratory and expiratory CT imaging. The physician could perform a biopsy by monitoring the location of the nodule according to the patient's respiratory cycle. In addition, the univariate analysis showed no statistical significance when the nodule movement was greater than the nodule size.

The complication rates were reported as 1%–2% for ENB‐related bleeding and 3%–5% for pneumothorax in previous studies.^12^, ^19^ Although our rate of pneumothorax was similar, the rate of bleeding was relatively higher in our study than in previous reports because we counted all bleeding events, including minor bleeding, unlike other reports. Prior studies reported a moderate bleeding rate of 2%,^23^, ^24^, ^25^ concordant with our result of 4%. Pneumothorax usually occurred when the biopsy was performed in an upper lobe area with severe emphysema.

This study had a few limitations. First, this was a retrospective study with ENB results determined by a single physician at a single center, so there may be selection bias. However, this study is valuable in that the ENB was performed in an Asian environment where most endoscopic procedures are done under moderate sedation, identifying the change in diagnostic yield and presenting a learning curve in this context. The diagnostic yield must be more accurately determined and the learning curve for ENB clarified by analyzing several operators’ experiences in various environments through future prospective multicenter research. Second, additional equipment, such as ROSE or R‐EBUS, was not used when performing ENB. Previous reports have indicated that the combination of R‐EBUS and ENB may improve the diagnostic yield.^24^, ^26^ In a study by Eberhardt et al.,^24^ the diagnostic yield of a combination of R‐EBUS and ENB for peripheral lung nodules was higher than that of ENB alone (88% vs. 59%; *p* = 0.02). Furthermore, using ROSE allows proper sample acquisition to be immediately confirmed during the biopsy, resulting in a diagnostic yield of more than 80%.^20^, ^27^ Further research must be conducted combining ENB with ROSE or R‐EBUS to develop the diagnostic yield of ENB. Third, most of the cases involved diagnostic testing for solid organ cancer, including lung cancer. Although this study included three cases of lymphoma, this number is insufficient to suggest the diagnostic yield of ENB for lymphoma with lung involvement. Although a diagnostic yield of more than 90% has been reported for EBUS in lung cancer, the diagnostic yield in lymphoma is relatively low (~70%).^28^, ^29^ In the case of ENB and EBUS, the diagnostic yield for lymphoma is presumed to be relatively lower than that for lung cancer because only very small tissues can be obtained from the peripheral airways. In our study, only lymphoid cells could be acquired in one lymphoma case, and we could not confirm the correct diagnosis through ENB biopsy. Therefore, further research on lymphoma diagnosis is necessary, such as for mucosa‐associated lymphoid tissue lymphoma, which only invades the pulmonary parenchyma. Further study on the diagnostic yield based on different biopsy devices, such as forceps or a needle, is also required.

In conclusion, ENB‐guided biopsy for high‐risk pulmonary nodules showed an acceptable diagnostic yield and a good safety profile. Moreover, the diagnostic yield was associated with tumor size and procedure experience. Considering that patients who require lung cancer screenings are often heavy smokers and have a greater possibility of underlying lung disease, it is important to make active use of ENB.

## AUTHOR CONTRIBUTIONS

Study concept and design: J.H.O., C‐M.C., and W.J. Data collection: J.H.O., C‐M.C., W.S.K., H.S.H., S.J.J., S.Y.O., M.Y.K., J.C.L., and W.J. Data analysis and interpretation: J.H.O., C‐M.C.,W. S.K., W.S.K., H.S.H., S.J.J., S.Y.O., M.Y.K., J.C.L., and W.J. Drafting of the manuscript: J.H.O. and W.J. Critical revision of the manuscript: C‐M.C. and W.J.

## DISCLOSURE

The authors declare that they have no competing interests to disclose.

## Supporting information


**Table S1** Diagnostic yield according to each variable
**Table S2** Diagnostic yield of nodules without bronchus sign according to biopsy device
**Table S3** Baseline characteristics of participants in the early and late phases
**Table S4** Baseline characteristics of pulmonary nodules in the early and late phases
