# Supplementary material for: Diagnostic yield and safety of biopsy guided by electromagnetic navigation bronchoscopy for high‐risk pulmonary nodules
Source: Thorac Cancer. 2021 Mar 21;12(10):1503–10. doi: 10.1111/1759-7714.13930 (PMC8107026; doi:10.1111/1759-7714.13930)
Supplement: Supplementary file 1 — Table S1 Diagnostic yield according to each variable Table S2 Diagnostic yield of nodules without bronchus sign according to biopsy device Table S3 Baseline characteristics of participants in the early and late phases Table S4 Baseline characteristics of pulmonary nodules in the early and late phases [file TCA-12-1503-s001.docx]

**Supplementary material**

**Table S1**. Diagnostic yield according to each variable

| Variable | Diagnostic yield, Number/Total (%) | p-value |
| --- | --- | --- |
| Upper lobe lesion | 27/49 (55.1) | 0.680 |
| Non-upper lobe lesion | 26/51 (51.0) |  |
| Nodule size ≥ 20 mm | 44/75 (58.7) | 0.049 |
| Nodule size < 20 mm | 9/25 (36.0) |  |
| Solid-type nodule | 3/5 (60.0) | 0.999 |
| Non-solid-type nodule | 50/95 (52.6) |  |
| Bronchus sign |  |  |
| Present | 43/71 (60.6) | 0.018 |
| Absent | 10/29 (34.5) |  |
| Bronchus sign localization |  |  |
| Concentric | 33/55 (60.0) | 0.857 |
| Eccentric | 10/16 (62.5) |  |
| Metabolic activity in PET, mean SUV_max_ ≥ 6 | 17/29 (58.6) | 0.778 |
| Metabolic activity in PET, mean SUV_max_ < 6 | 26/47 (55.3) |  |
| Distance from Visceral pleura ≥ 10 mm | 22/51 (43.1) | 0.044 |
| Distance from Visceral pleura < 10 mm | 31/49 (63.3) |  |
| Total procedure time ≥ 20 min | 12/33 (36.4) | 0.015 |
| Total procedure time < 20 min | 41/66 (62.1) |  |
| Biopsy device |  |  |
| Forceps only | 33/63 (52.4) | 0.871 |
| Needle or forceps + needle | 20/37 (54.1) |  |

Data are presented as number (%)

**Table S2**. Diagnostic yield of nodules without bronchus sign according to biopsy device

|  | Diagnostic yield, Number/Total (%) | p-value |
| --- | --- | --- |
| Total nodule without bronchus sign | 10/29 (34.5) |  |
| Biopsy device |  |  |
| Forceps only | 6/16 (37.5) | 0.999 |
| Forceps + needle | 4/13 (30.8) |  |

Data are presented as number (%)

**Table S3**. Baseline characteristics of participants in the early and late phases

|  | **1-60 cases** | **61-100 cases** | **p-value** |
| --- | --- | --- | --- |
| Total participants | 54 | 37 |  |
| Age in years, median (range) | 64.5±11.6 | 67.8±9.1 | 0.147 |
| Male sex | 32 (59.3) | 24 (64.9) | 0.589 |
| Ever smoked | 29 (53.7) | 20 (54.1) | 0.974 |
| Pulmonary function (n = 81) |  |  |  |
| FVC, % pred, mean ± SD | 87.4±15.1 | 84.3±12.5 | 0.319 |
| FEV1, % pred, mean ± SD | 84.3±20.3 | 76.2±20.3 | 0.076 |
| DLco, % pred, mean ± SD | 76.0±19.8 | 72.7±30.1 | 0.573 |
| Cause of ENB (n cases = 100) |  |  |  |
| Emphysema | 9 (15) | 7 (17.5) |  |
| Vascular structure | 43 (71.7) | 20 (50) |  |
| Inaccessible | 2 (3.3) | 5 (12.5) |  |
| No diagnosis by PCNB | 5 (8.3) | 2 (5) |  |
| At high-risk of pneumothorax and bleeding | 1 (1.7) | 6 (15) |  |
| Intravenous sedation | 54 (100) | 37 (100) |  |
| ENB procedure time, mean ± SD, min | 14.2±6.5 | 9.9±3.7 | <0.001 |
| Total procedure time, mean ± SD, min | 18.1±7.5 | 16.4±6.1 | 0.254 |

Data are presented as number (%), mean ± standard deviation (SD), or median (interquartile range); FVC, forced vital capacity; FEV_1_, forced expiratory volume in one second; DLco, diffusing capacity of the lung for carbon monoxide; PCNB, percutaneous needle biopsy; IV, intravenous

**Table S4.** Baseline characteristics of pulmonary nodules in the early and late phases

|  | **1-60 cases** | **61-100 cases** | **p-value** |
| --- | --- | --- | --- |
| Total nodules | 60 | 40 |  |
| Upper/middle lobe location | 35 (58.3) | 23(57.5) | 0.934 |
| Size, mm ± SD | 26.0±11.8 | 30.8±15.9 | 0.082 |
| Solid type | 55 (91.7) | 40 (100) | 0.081 |
| Bronchus sign present | 40 (66.7) | 31 (77.5) | 0.242 |
| Concenteric | 33 (82.5) | 22 (71.0) | 0.249 |
| Eccenteric | 7 (17.5) | 9 (29.0) |  |
| Metabolic activity in PET, mean SUV_max_ ± SD (n = 76) | 4.9±3.6 | 7.5±6.3 | 0.037 |
| Distance from visceral pleura, mm ± SD | 15.3±14.2 | 11.2±9.9 | 0.092 |
| Biopsy tools |  |  |  |
| Forceps only | 31 (51.7) | 32(80.0) | 0.004 |
| Needle or forceps + needle | 29 (48.3) | 8 (20.0) |  |
| Number of biopsy attempts per lesion | 6.6±2.3 | 6.7±1.7 | 0.939 |

Data are presented as number (%), mean ± standard deviation (SD); SD, standard deviation
